# Legal Notes for Institutional Workers

**Published:** 1912-05-18

**Authors:** 


### LEGAL NOTES FOR INSTITUTIONAL WORKERS.

(The Editor will be pleased to answer Legal queries in this column.)

### Hospitals and the Shops Act.

The question whether and how far the Shops Act, 1911, which has recently come into force is applicable to hospitals is bound to agitate the mind of the institutional manager sooner or later. What of the hospital dispensary? Is it a shop within the meaning of the Act? According to section 14 of the Act the expression "shop" includes any premises where any retail trade or business is carried on, while the expression "retail trade or business" includes the business of a barber or hairdresser, the sale of refreshments or intoxicating liquors, and retail sales by auction, but does not include the sale of programmes and catalogues and other similar sales at theatres and places of amusement." The operative part of the Act provides that on at least one week-day in each week a shop assistant shall not be employed about the business of a shop after half-past one o'clock in the afternoon. It also provides that every shop shall, save as otherwise provided by this Act, be closed for the serving of customers not later than one o'clock in the afternoon of one week-day in every week.

### Is the Hospital Dispenser a Shop Assistant?

As to the compulsory half-holiday to shop assistants, it would seem that if the dispenser at a hospital dispensary is a shop assistant he must have his half-holiday. The expression "shop assistant" means any person wholly or mainly employed in a shop in connection with the serving of customers or the receipt of orders or the despatch of goods. It is extremely doubtful whether a dispenser would come within this definition. With reference, however, to the compulsory closing once a week, we think that institutional managers need be under no apprehension. This highly domestic piece of legislation does not apply to certain trades or businesses which are enumerated in the Second Schedule to the Act, amongst which are: "The sale of medicines and medical and surgical appliances." It is, however, provided that a local authority may by order eschew the provision which prescribes a compulsory half-holiday to shops of any exempted class if satisfied that the occupiers of at least two-thirds of the shops of that class approve the order. It would seem to follow that the local authority has it within its power to close the chemists' shops, but the hospital dispensaries are not so numerous in any district that they could be magnified into a "class," nor would they be likely to prefer a petition for early closing.

### The Collecting of Debts from Patients.

The doctor who assigns a bad debt to a collector saves the time which he would otherwise spend in attending at the local county court. But the debt collecting agency may be a trap for the unwary. In a case heard at the Wandsworth County Court last week, Dr. Ritchie, of Cavendish, Suffolk, brought suit against the "South-West London Tradesmen's Provident Society, Ltd.," to recover the sum of £4 4s. In reality, the business was run by two gentlemen named Cook and Rackham. Those who paid a guinea a year to this body were apparently entitled to benefits of no mean order. "Advice on any matter free of charge; solicitor's advice on all legal matters; the retaining of eminent counsel; private companies formed; mortgages a speciality." These were some of the attractions. There was also a debt collecting department. The plaintiff, being owed £17 19s. 6d. in small sums, requested the society to collect them. He received no answer, however, until March 17, 1911, when the society admitted having collected £4 4s. The defendants, a day or two before the hearing, paid £4 4s. and costs into court. Counsel for the plaintiff said that the action had been brought with the assistance of the Medical Defence Union with a view to protecting the medical profession.

